# The Role of Liquid Based Cytology and Ancillary Techniques in the Peritoneal Washing Analysis: Our Institutional Experience

**DOI:** 10.1371/journal.pone.0168625

**Published:** 2017-01-18

**Authors:** Esther Rossi, Tommaso Bizzarro, Maurizio Martini, Adhemar Longatto-Filho, Fernando Schmitt, Anna Fagotti, Giovanni Scambia, Gian Franco Zannoni

**Affiliations:** 1 Division of Anatomic Pathology and Histology - Università Cattolica del Sacro Cuore, “Agostino Gemelli” School of Medicine, Rome, Italy; 2 Department of Pathology, University of São Paulo School of Medicine, Sao Paolo, Brazil; 3 Life and Health Sciences Research Institute (ICVS), School of Health Sciences, University of Minho, Braga, Portugal; 4 ICVS/3B’s Laboratory of Medical Investigation (LIM)- PT Government Associate Laboratory, Braga/Guimarães, Portugal; 5 Molecular Oncology Research Center, Barretos Cancer Hospital, Pio XII Foundation, Barretos, Brazil; 6 Medical Faculty, Porto University, Porto, Portugal; 7 Laboratorie National de Sante, Luxembourg, Luxembourg; 8 Gynecological Oncology, Catholic University, Rome, Italy; Queen Mary Hospital, HONG KONG

## Abstract

**Background:**

The cytological analysis of peritoneal effusions serves as a diagnostic and prognostic aid for either primary or metastatic diseases. Among the different cytological preparations, liquid based cytology (LBC) represents a feasible and reliable method ensuring also the application of ancillary techniques (i.e immunocytochemistry-ICC and molecular testing).

**Methods:**

We recorded 10348 LBC peritoneal effusions between January 2000 and December 2014. They were classified as non-diagnostic (ND), negative for malignancy-NM, atypical-suspicious for malignancy-SM and positive for malignancy-PM.

**Results:**

The cytological diagnosis included 218 ND, 9.035 NM, 213 SM and 882 PM. A total of 8048 (7228 NM, 115SM, 705 PM) cases with histological follow-up were included. Our NM included 21 malignant and 7207 benign histological diagnoses. Our 820 SMs+PMs were diagnosed as 107 unknown malignancies (30SM and 77PM), 691 metastatic lesions (81SM and 610PM), 9 lymphomas (2SM and 7PM), 9 mesotheliomas (1SM and 8SM), 4 sarcomas (1SM and 3PM). Primary gynecological cancers contributed with 64% of the cases. We documented 97.4% sensitivity, 99.9% specificity, 98% diagnostic accuracy, 99.7% negative predictive value (NPV) and 99.7% positive predictive value (PPV). Furthermore, the morphological diagnoses were supported by either 173 conclusive ICC results or 50 molecular analyses. Specifically the molecular testing was performed for the *EGFR* and *KRAS* mutational analysis based on the previous or contemporary diagnoses of Non Small Cell Lung Cancer (NSCLC) and colon carcinomas. We identified 10 EGFR in NSCCL and 7 KRAS mutations on LBC stored material.

**Conclusions:**

Peritoneal cytology is an adjunctive tool in the surgical management of tumors mostly gynecological cancers. LBC maximizes the application of ancillary techniques such as ICC and molecular analysis with feasible diagnostic and predictive yields also in controversial cases.

## Introduction

The role of the analysis of peritoneal washing, mostly employed in gynecological diseases, has not been completely defined in its entire diagnostic and prognostic potentialities [1–5].

A general evaluation of body effusions by Lee et al underlined that one-fifth of body serous membrane effusions per year are malignant with about 50% as metastatic adenocarcinomas followed by pulmonary large cell carcinomas and lymphomas/leukaemia (about 15% each) [1–3]. It stands to reason that the diagnostic detection of malignant cells in effusions serves as a mainstream diagnostic tool and a predictor of the spread of diseases with impact on prognosis and upstages prognostic morbidity. Hence, the routinely cytological preparations represent a simple, safe and cost-effective method limiting the amount of issues associated with biopsy procedures, which often result in non-diagnostic histological results [1–5]. Specifically, Creasman and Rutledge, reported that the majority of positive peritoneal washings are associated with gynecological cancers (the highest percentage of positive washings was in ovarian followed by endometrial and rarely cervical cancers); so that their cytological evaluation may stratify patients in either the initial diagnosis of symptomatic gynecological cancers or the management and survival [6]. In fact, since the 1950s, peritoneal washing has been an essential part of the management of ovarian and endometrial carcinomas with the exception of cervical cancers [7–15]. Despite this consideration, the 2008 revision of the International Federation of Gynecology and Obstetrics (FIGO) eliminated the performance and analysis of washing yields from the endometrial carcinoma staging. The reasons are due to both the criticism emphasized in several studies and the lack of cytological criteria.

One of the most problematic dilemmas is the discrimination between malignant cells (including metastatic cells) and reactive mesothelial cells because the lack of specific cytological criteria for malignancy may lead to possible diagnostic pitfalls [16–20]. In this perspective, it is notable that the applications of ancillary techniques support the diagnostic stratification of the risk of malignancy [16–31]. Even though the majority of published reports demonstrated good results with each of the cytological techniques, we focused on our 14-year experience with the largest series of peritoneal cytological samples processed by liquid based cytology (LBC) and compare our results with the literature data [32–35]. Furthermore, we analyzed the role of ancillary techniques (ICC and molecular analysis) as an additional tool in peritoneal LBC samples.

## Materials and Methods

We included all the 10348 cytological samples of peritoneal washings analyzed in the period between January 2000 and December 2014. All the cases were recorded in the Division of Anatomic Pathology and Histology of the Catholic University, “Agostino Gemelli” Hospital of Rome (Italy). All cytological washings were carried out mostly by surgeons and processed with Thin Prep 5000^™^ method (Hologic Co., Marlborough, MA). All the data were analyzed in details in the result section. We focused our specific attention and discussion on the suspicious and positive neoplastic series.

The series included 1867 male and 8481 female patients with a median age of 48 years (range 23–92 y/o). During the surgical procedures, all the incidental free fluid was aspirated and if free fluid is not present, 10–100 ml saline (0.9%) natriumchloride solution) was introduced to lavage the peritoneal cavity. No rapid on-site evaluation of the adequacy of the material was done. All patients had been appropriately informed regarding the use of LBC method for processing their samples and a written informed consensus were signed. Our study followed the tenets of the Declaration of Helsinki and we received the internal department (Department of Anatomic Pathology and Histology) ethics approval for the study. We need to specify that we followed the general and normal ethic statement of our University (Catholic University) as long as this is an immunocytochemical study used for diagnostic purposes which do not need any ethical university reviewer board approving the study in our institution but only the one from our department.

All the material was fixed with the hemolytic and preservative solution Cytolyt^™^ after rinsing the needle in this solution. The cells were spun at 1500 rpm (rotations per minute- 0,289 xg); then, the sediment was transferred in the Preservcyt^™^ solution to be processed with the T2000 automated processor according with the manufacturer`s recommendation. The resulting slide was fixed in 95% ethanol and stained with Papanicolaou while the remaining material was stored in the Preservcyt^™^ solution to be possibly used for the preparation of additional slides for further investigations (including both ICC and molecular analysis).

Herein the definition of the morrpholoical criteria: Cytological diagnoses were primarily considered as “adequate for a cytological diagnosis “or “inadequate for a cytological diagnosis”. The former group was sub-classified into: 1) Negative for malignancy; 2) atypical- Suspicious for malignancy; 3) Positive for malignancy. We also signed out cases with “positive for malignancy supporting/ confirming the primary cancer diagnosis.

Additional slides for ICC (68 malignancies and all 105 atypical suspicious cases) and/or molecular analysis (50 cases) were obtained from the material stored in the PreservCyt solution; so, that they can be performed with even 2 ml remaining material eluted in 5 ml of PreservCyt solution.

The percentage of disease specific cells for immunocytochemical (ICC) analysis was at least 30% in all LBC samples.

### Immunocytochemistry

ICC stainings were carried out with the avidin-biotin peroxidase complex using a variety of antibodies based on both the clinical suspects and our cytological findings. The slides were washed 3 times in phosphate-buffered saline (PBS) and then pre-incubated in normal veal serum with PBS (1:50) for 20 minutes before overnight incubation at 4°C with the primary antibody. Then, the slides were washed 3 times with PBS and incubated with the biotinylated secondary antibody conjugated with the avidin-biotin-peroxidase complex (Ventana, USA). The reaction was developed using 3–3’ diamino-benzidine (DAB) as a chromogen. All slides were counterstained with hematoxylin for 5 seconds, rinsed in water 3 times and then mounted for the microscopic examination. The positivity was assessed, for each cytological case, when at least 50% of cells showed a strong cytoplasm or nuclear positivity based on the specific immunomarker used. This stringent cut-off percentage was chosen in order to avoid false positive results and lined with the reported stained data on the histological diagnoses. Positive controls and negative controls were selectively used in agreement with each specific immunomarker. Our ICC evaluation was carried out in 173 (22%) out of 820 SM + PM cases and specified for the different immunomarkers

### Molecular analysis

According to our previous studies, genomic DNA was extracted from cytological samples using a spin column extraction method (QIAamp DNA mini kit; QIAGEN, Milan, Italy). DNA concentration and purity were assessed using NanoDrop 2000c Spectrophotometer (Thermo Scientic Inc, Wilmington, DE, USA). Mutational analysis of *EGFR* and *KRAS* was performed using the therascreen *EGFR and KRAS* RGQ PCR Kit (QIAGEN) in Rotor-Gene Q 5plex HRM instrument, following the manufacturer’s protocol (sensitivity <1%). The mutation nomenclature used in this work follows the guidelines indicated by Human Genome Variation Society [36].

### Histology

The cases with surgical specimens were fixed in 10% buffered formaldehyde, embedded in paraffin and the 5-micron-thick sections were stained with Haematoxylin-Eosin. The concordance of ICC between the cytological and histological samples was 99.97% with three discrepancies out of 8048 cases with surgical follow-up (including 7228 NM, 115 SM and 705 PM).

### Statistical analysis

Statistical analysis was performed by using a commercially available statistical software package (SPSS 10.0, Chicago, IL, USA) for Windows (Microsoft, Redmond, Washington, USA). Comparison of categorical variables was performed by chi-square statistic, using the Fisher’s exact test when appropriate. A *p* value less than 0.05 were considered significant.

## Results

The study cohort included 10348 cases between January 2000 and December 2014. The clinicopathological data including all the cytological diagnoses were summarized in Table 1.

**Table 1:** Clinico-morphological evaluation in 10348 peritoneal effusions in LBC.

	NM	SM	PM
**Age**	23–80	24–86	31–90
**Sex (M/F)**	1529/7506	72/141	167/715
**Cell Block Additional Slides**	582	104	356
**Histology**	7228	115	705
**ICC/Molecular tests**	0	105/9	68/41

M: male; F: female; ICC: immunocytochemistry; NM: Negative for Malignancy; SM: Suspicious for Malignancy; PM: Positive for Malignancy. Our 218 Non-diagnostic cases are excluded

The cohort included 8481 female and 1867 male patients with age ranging from 19 to 90 years (mean: 54 years) showing a significantly higher female distribution.

The cases were distributed based on their diagnoses as non-diagnostic (ND), negative for malignancy (NM), atypical-suspicious for malignancy (SM) and positive for malignancy (PM). Specifically the series exhibited 218 ND, 9.035 NM samples; 213 SM, and 882 PM [Table 1]. We did rule out the 218 ND cases because they remained ND even after having performed an additional slide.

The 9.035 NM did not show any false positive results but 21 false negative results. The NM category was characterized by 7.228 cases with a histological diagnosis including 6459 NM with a histological diagnosis of cancer without peritoneal involvement, 748 NM with benign histology and 21 false negative cases. Hence, the majority of cases had only Thin Prep (TP) slides (7260 cases), whilst 1775 had an additional cell block in order to combine the LBC slide and cell block for a more accurate morphological diagnosis. In none of these NM cases we carried out any immunomarker or molecular testing including also the 21 false negative cases which were associated with histological specimens on which we performed a conclusive ICC analysis (Table 1).

**Fig 1:**
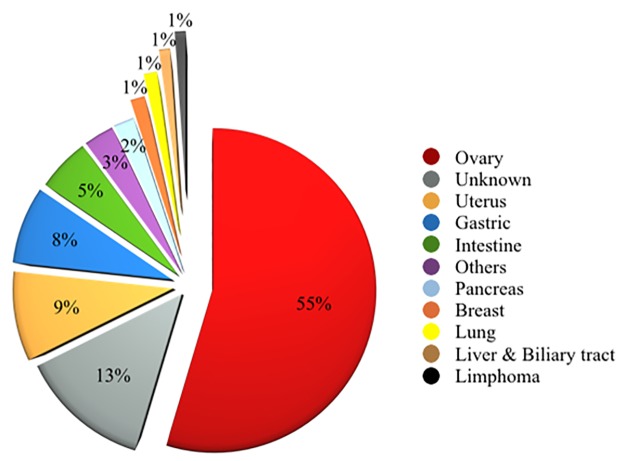
Diagram summarizing the data concerning Suspicious for malignancy (SM) plus Positive for malignancy (PM) peritoneal cases.

**Fig 2:**
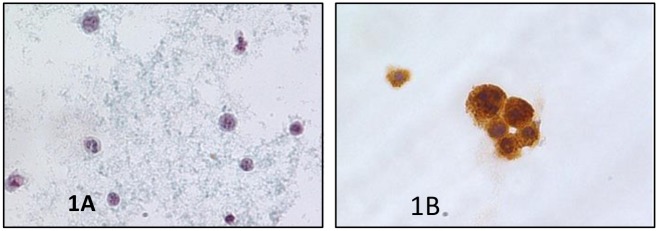
a) Details of a malignant peritoneal effusion from an ovarian carcinoma showing a papillary architecture of a neoplastic cellular cluster (PAPx400, LBC); b) Positivity for CA125 in the same Fig 1 (peritoneal effusion from an ovarian carcinoma) on LBC (avidin-biotin-peroxidase complex x400, LBC).

**Fig 3:**
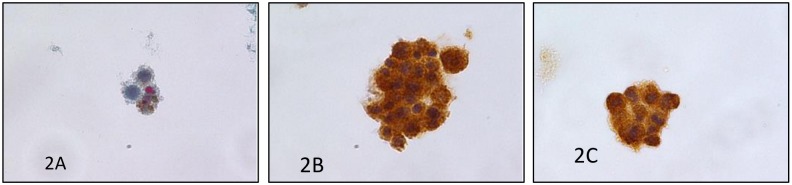
a) Details of a malignant peritoneal effusion from a metastatic melanoma showing the pleomorphic features and pigment (PAPx400, LBC); b) Positivity for S100 in the same case (peritoneal effusion from a metastatic melanoma) on LBC (avidin-biotin-peroxidase-ABC complex x400, LBC); c) positivity for Melan A in the same case (ABC complex x400, LBC).

The group of 1.095 SM plus PM categories reported 820 cases (115 SM and 705 PM) with histological follow-up; the distribution of the different malignancies was detailed in Table 2 and Fig 1. In these categories, we found two false positive cases belonging to the SM category. Furthermore, as shown in the table, gynecological cancer represented the most frequent metastatic cancer including 448 ovarian (Fig 2a), 74 uterine and three tubaric-ovarian carcinomas. They were followed by gastro-intestinal carcinomas distributed as 64 gastric, 44 intestinal, 17 pancreatic and 8 biliary tract carcinomas. All these patients had a preceding and/or contemporary histological diagnosis of malignant diseases (Fig 3a).

**Table 2:** Distribution of peritoneal diagnoses with histological findings.

Histology	Cytological Diagnoses				
	Inadequate	NM*(FN)	SM	PM	Ancillary Technique ICC/MT
**Unknown**	0	43 (3)	30	77	27/0
**Lung Ca**	0	39 (1)	2	9	7/11
**Ovarian + Tubaric Ca**	0	6739 (7)	36	415	72/0
**Uterine Ca**^§^		107 (0)	11	63	11/0
**Breast Ca**	0	72 (0)	1	11	2/0
**Gastro-Intestinal Ca**	0	173 (4)	23	85	27/39
**Liver + biliary tract+ pancreas Ca**		27 (3)	7	19	11/0
**Mesothelioma**	0	3 (2)	1	5	7/0
**Melanoma**	0	0 (0)	0	4	4/0
**Kidney/Urological Ca**	0	0 (0)	1	4	4/0
**Lymphoma**	0	4 (1)	2	7	1/0
**Sarcoma**	0	0 (0)	1	3	0/0
**Primary peritoneal Ca**	0	0 (0)	0	3	0/0

Ca: Carcinoma; ICC: Immunocytochemistry; NM: Negative for Malignancy; SM: Suspicious for Malignancy; PM: Positive for Malignancy; ICC: immunocytochemistry; MT: Molecular testing

*including the 21 false negative (FN) cases and the 7207 with malignant histology without peritoneal involvement. We did not report the 748 NM with benign histology. Hence, none of the 21 false negative cases had ICC;

^§^ all the cases were endometrial carcinomas

In the same Table 2, Fig 1 and then detailed in Table 3, we analyzed the application of ICC, which was performed as immunopanel rather than single immunomarker. We found that a total of 173 effusions (all the 105 SM and 68 PM) carried out ICC on the TP stored material, with 100% irrefutable results. ICC was performed on both TP stored slide and cell block obtained from TP stored material without any significant difference in the ICC expression.

**Table 3:** Correlation between the primary tumors and the peritoneal effusions: Morphological and ICC profiles.

Primitive Cancers		SM/PM (105/68)	Antibody Tested On Cytological And Cell Block Samples°
**Unknown (27 cases)**		**20/7**	Positive*: 1 Vimentin;7 Calretinin, 1 TTF-1; 6 CEA; 20 CAM 5.2M; 7 BER-EP4; 2 CK7, 3 CK20, 1 WT1, 4 CD68
**Breast Ca (2 cases)**		**0/2**	**Positive:** CK7;GCDFP15;ER;PR;E-Cadherin **Negative:** TTF-1, Calretinin, CK5/6.
**Lung Ca (7 cases)**		**2/5**	**Positive:**TTF-1;CAM5.2;CK7; p63. **Negative:** S100, Calretinin, CK5/6
**Gynecological Ca (83 cases)**			
	**• Ovarian Ca (71 cases)**	**36/35**	**Positive:** CA125, WT1,CAM 5.2, CK7, ER, PR, CEA. **Negative:** S100,CK5/6, Calretinin, CK20, CDX2
	**• Endometrial Ca (11 cases)**	**11/0**	
	**• Tubaric-ovarian Ca (1 cases)**	**1/0**	
**Mesothelioma (6 cases)**		**3/3**	**Positive:** CAM 5.2, Calretinin, HBME-1, Podoplanin. **Negative:** TTF-1
**Gastro-Intestinal Ca (39 cases)**			
	**• Stomach-Intestinal Ca (27 cases)**	**24/3**	**Positive:** CAM 5.2;CK20; CDX2. **Negative:** TTF-1, Calretinin, CK5/6.
	**• Biliary Ca (6 cases)**	**3/3**	
	**• Pancreatic Ca (6 cases)**	**3/3**	
	**• Liver Ca (0 cases)**	**0/0**	
**Lymphoma (1 case)**		**1/0**	**Positive:** LCA, CD20, CD79A, CD30, CD15. **Negative:** TTF-1,CAM 5.2;CK20; CDX2, CK5/6, Calretinin.
**Renal-Urogenital Ca (4 cases)**		**1/3**	**Positive:** EMA;CAM 5.2;Vimentin, CD10. **Negative:** Thyroglobulin; TTF-1.
**Melanoma (4 cases)**		**0/4**	

Ca: Carcinoma;

* we reported the figures for positive results so that the remaining figures resulted as corresponding negative value;

° ICC was performed on both LBC stored slide and Cell block obtained from LBC stored material without any significant difference in the expression.

In Table 3 we showed the distribution of immunomarkes in our SM and PM effusions (Figs 2b, 3b and 3c)

In fact we carried out an extensive panel made up of epithelial markers (i.e. Keratins 7 and 20, AE1/AE3, CAM 5.2, ESA, Thyroid trascriptor factor-1 [TTF-1], E-cadherin, and others detailed in Table 3), mesenchymal markers (Vimentin, Desmin, neurofilaments) CD markers (CD10, CD15, CD30, CD45) or other markers (S100, mCEA, NSE, Calcitonin, HMB45).The ICC panels were carried out in both unknown primary tumors and in cases with a clinical neoplastic history but controversial cytological samples.

The evaluation of specificity, sensitivity, and diagnostic accuracy, positive (PPV) and negative (NPV) predictive values was worked out including the two false positive cases in the SM category and the 21 false negative cases in the NM. We documented 97.4% sensitivity, 99.9% specificity, 98% diagnostic accuracy, 99.7% negative predictive value (NPV) and 99.7% positive predictive value (PPV). Moreover, we had 50 PM effusions tested for the *EGFR* and *KRAS* mutational analysis based on the previous or contemporary diagnoses of Non Small Cell Lung Cancer (NSCLC) and colon carcinomas. We found 10 *EGFR* mutated NSCLC (6 with short in-frame deletions of exon 19 and 4 cases with single-nucleotide substitutions in exon 21 characterized by the missense mutation p.L858R); and, 7 *KRAS* mutated colon carcinomas [data not tabled].

## Discussion

The analysis of peritoneal washings, herein reported, has demonstrated the prominent role of cytology processed by LBC slides. As stated by literature, cytological method is striving to gain more diagnostic yields from effusion samples mainly due to the difficulties in the discrimination between benign and malignant disease and between benign reactive mesothelial cells and adenocarcinoma or mesothelioma [16–21]. Indeed, despite the fact that the regular sheet-like arrangements of benign mesothelial cells make an easy identification and discrimination in the majority of cases, large folded sheet of mesothelial cells can mimic a malignant appearance [13]. Equally important, the evidence of some pleomorphic features cannot make discrimination between borderline and low-grade tumors challenging [13]. Nonetheless, some findings, including cilia, clusters of cells, lack of isolated cells, scant cytoplasm vacuolation and absence of mitotic activity support a benign diagnosis. In spite of the acknowledge that the majority of positive peritoneal dissemination is associated with gynecological malignancies, particularly ovarian tumors, there is the evidence that other malignancies, such as gastro-intestinal carcinomas, pancreatic and cholangiocarcinoma are found with significant prevalence in peritoneal effusions [6–10]. The relevance of the role of peritoneal cytology in diagnosing malignant spread, especially in advances diseases, is also demonstrated by the huge impact, since the 1950s, that peritoneal cytology has been an integral component of the management and prognosis of patients with ovarian and endometrial carcinomas [6]. Hence, this evidence is still supported by the fact that surgeons send peritoneal washing for cytological evaluation in contrast with the statements proposed by 2008 revised FIGO guidelines. In fact, these cytological yields are used to monitor response to chemotherapy, to identify microscopic spread of tumor cells and exclude occult cancer in benign entities. However, the morphological analysis is not able to clarify all the doubts and answer about all the diagnostic queries. Allegedly, as highlighted by Ylagan et al, some morphological overlaps prevent from a conclusive diagnosis and they maximize the need for the selection of a panel of immunomarkers [17]. Several papers highlighted the improvements in the sensitivity and specificity of peritoneal washings from gynecological and gastro-intestinal cancers using a wide range of markers to define either a primary or a metastatic origin [20–23]. This adjunct seems to be empowered by the use of a panel of immunomarkers that is more easily applied when LBC is adopted [1–4]. The well-known controversial data concerning the efficacy of LBC are counter-parted by the reliable and feasible yields, including cost-effectiveness; time-sparing and easy application of ancillary techniques up to 3–4 months on the LBC stored material [1–5, 24–26, 30, 32, 34]. Besides, an article by Hoda evaluated all the known literature for the morphological aspects of LBC in non-gynecological cytology as well as the accuracy of both TP and SurePath in the interpretations of effusions in alignment with the data from Ylagan et al [3, 17].

The introduction of LBC, as and alternative preparation for cytological specimens, offers the possibility to shift from the morphological cellular details to the prognostic significance and predictive value of their characterization through the support of ancillary techniques. Not only did LBC offer some additional morphological advantages (clearer background, cell enrichment and better nuclear details) but also LBC residual material seems to be useful for the application of immunocytochemical markers but also, and equally important, for molecular tests [1–5, 24–26, 30, 32, 34]. That is a central point to be highlighted because LBC diagnoses are comparable to those obtained with traditional smears and offer an additional value in order to carry out the analysis of receptor gene rearrangements by polymerase chain reaction (PCR) or chromosome translocation by fluorescence in situ hybridization (FISH).

In the current analysis of 10348 peritoneal effusions processed with TP method, 93% of cases were benign lesions and 7% malignant, emphasizing the established fact that the majority of peritoneal effusions resulted in benign diagnoses. Moreover, our 99.8% benign and 99.6% cyto-histological correlation demonstrated a higher value than that reported by literature mostly due to the application of TP preparation [6–15].

That said, in our experience, the evidence of only 21 false negative cases (0.002%) pointed to the high negative predictive value of the liquid based cytological preparation. In fact, in the majority of our cases we performed a second TP slide and cell block obtained from TP stored material; both of them were concordant also with the corresponding histological samples on which we perform ICC.

Another interesting point of discussion is the analysis of our inadequate rate. Our results documented that the low (2.1%) non-diagnostic rate is one of the advantage of the additional TP slides and cell blocks with a consequent decreasing inadequate rate. Likewise, the 218 non-diagnostic samples were devoid of adequate material so as not to carry out the preparation of a second TP slide or cell block and application of any ancillary techniques. However, according to Shield, despite the significant improvement in the diagnostic adequacy, it stands to reason that also LBC method cannot obtain 100% conclusive diagnoses resulting in minimal influence on clinical and surgical management [13].

In the current paper, the presence of 213 suspicious for malignancy cases emphasized the consciousness that there are some “critical” morphological findings, which overlap between reactive mesothelial cells and mesothelioma even among experience cytopathologists [16–21]. In our experience, diagnostic concordance was achieved between TP and histological diagnoses in 114 out of 115 SM cyto-histological cases with the exception of one SM resulted in a benign peritoneal histology. This yield revealed the clinical validity and high risk of malignancy for this diagnostic category. Thus, attesting to the fact that good to excellent sensitivity and predictive value can be achieved for peritoneal cytological specimens processed with TP.

We reported 10.6% malignant rate, which is in perfect agreement with the 15% malignancy reported by Lee et al on LBC whilst the discrepancies with other series are a consequence of the different preparations and the cyto-histological biases.[1, 6–13].

In keeping with the data by literature, our yields established that the majority of peritoneal effusions represent metastatic involvement from primary adenocarcinoma particularly in female patients with gynecological carcinomas whilst gastro intestinal carcinoma in both sexes [6–15]. According to Ditto et al, we did not find any case of positive effusions in squamous cervical carcinoma [15]. In detail, in our peritoneal effusions series we diagnosed 64% gynecological metastatic cancers including 55% ovarian and 9% endometrial carcinomas, followed by gastro-intestinal cancers (16%) but also 13% of unknown primary origin. Even though we focused on the central role of morphology on FNAC, we drew our attention to carry out the most specific useful ICC panel unless we were testing cases with unknown malignancies in which a large number of markers were carried out. The enthusiasm for the application of ancillary techniques, emphasized by several studies is also correlated with our 48% conclusive ICC in the gynecologic cases and 25.2% in the unknown malignant effusions. Moreover, the application of ICC was likely to establish a conclusive diagnosis in the majority of our SM demonstrating its invaluable diagnostic aid.

In fact, 21% of the suspicious and malignant effusions were supported by ICC application including specific diagnostic immunomarkers with 27 unknown primary neoplasm samples tested with several immunomarkers and resulting in 100% conclusive results without any technical issues. Additionally, we also tested 50 cases for the analysis of molecular testing on effusion based on our recent experiences proving that TP method does not affect DNA or RNA quality. We estimated *EGFR* mutational expression (including all mutations from exon 18 to 21) and *ALK* rearrangement on 10 (34%) metastatic effusions in NSCLC, and 7 *KRAS* genes in intestinal metastatic peritoneal carcinomas. These additional gene mutational assays corroborate the outstanding role of cytology to confirm malignancy but also to add prognostic parameters to the patient outcome. Furthermore, during these last years, it has been shown that malignant tumors are driven by specific somatic mutations including gene fusion, which can be studied by parallel RNA sequencing as well as whole-exome gene sequencing even on cytological material including peritoneal effusions [23, 27–30]. Nonetheless, further evaluation of these ancillary techniques must be judiciously investigated in large cytological cohorts to ascertain their applicability in the diagnosis of cytological effusion and to reduce its shortcoming

Eventually and in agreement with literature, the statistical analysis, based on histo-pathologic correlation, showed 97.4% sensitivity, 99.9% specificity, 98% diagnostic accuracy, 99.7% negative predictive value (NPV) and 99.7% positive predictive value (PPV), which undoubtedly exceeded the average value underscored in several series performed with conventional/cytospin-based cytology (7–11). In conclusion, to date, this is the largest series of peritoneal effusions with TP preparation and based on these figures we assessed that morphology represents a valid support especially in gynecological carcinomas. However, the application of both ICC and molecular analysis on LBC may prove to be an additional strategy, particularly in non-univocal diagnosis and in cases diagnosed as suspicious for malignancy in which a histological sample cannot be obtained.
